# Artificial intelligence‐guided assessment of the hip‐knee‐ankle angle shows excellent correlation with experienced human raters

**DOI:** 10.1002/jeo2.70383

**Published:** 2025-08-27

**Authors:** Mikhail Salzmann, Robert Prill, Roland Becker, Andreas G. Schreyer, Simon Shabo, Nikolai Ramadanov

**Affiliations:** ^1^ Center of Orthopaedics and Traumatology, Brandenburg Medical School University Hospital Brandenburg an der Havel Brandenburg an der Havel Germany; ^2^ Faculty of Health Science Brandenburg Brandenburg Medical School Theodor Fontane Brandenburg an der Havel Germany; ^3^ Institute for Diagnostic and Interventional Radiology Brandenburg Medical School Theodor Fontane Brandenburg an der Havel Germany

**Keywords:** artificial intelligence (AI), deep learning, hip‐knee‐ankle (HKA) angle

## Abstract

**Purpose:**

The hip‐knee‐ankle angle is a crucial parameter in orthopaedic surgery for lower limb assessment. However, traditional methods for measuring the hip‐knee‐ankle angle on standing long‐leg anteroposterior radiographs are time‐consuming, require significant expertise and lack reproducibility. Given the emergence of artificial intelligence as a promising tool to automate these measurements, this study aimed to assess the accuracy of Gleamer BoneMetrics for hip‐knee‐ankle angle measurement and its correlation with assessments by experienced orthopaedic surgeons.

**Methods:**

A total of 75 patients who underwent knee arthroplasty between October 2023 and June 2024 were included. Preoperative and postoperative long‐leg anteroposterior radiographs were analysed both by two experienced orthopaedic surgeons who manually measured the hip‐knee‐ankle angle and by the Gleamer BoneMetrics software; the analyses were tested for both inter‐ and intra‐rater reliability. Statistical analysis was performed using intraclass correlation coefficients and Bland‐Altman plots to assess the correlation between the Gleamer BoneMetrics and the human raters.

**Results:**

The Gleamer BoneMetrics demonstrated excellent inter‐ and intra‐rater reliability, with intraclass correlation coefficient values ranging from 0.995 to 0.998, which were comparable to the surgeons' measurements of 0.997–0.998. The Gleamer BoneMetrics's intra‐rater reliability was also excellent, with intraclass correlation coefficient values of 1.0 preoperatively and 0.996 postoperatively. Bland–Altman analysis showed minimal measurement discrepancies between Gleamer BoneMetrics and the human raters. However, in 2% of the cases (*n* = 3), Gleamer BoneMetrics was not able to provide measurements.

**Conclusion:**

The artificial intelligence‐based BoneMetrics software offers an efficient and accurate method for hip‐knee‐ankle angle measurement, with performance comparable to experienced orthopaedic surgeons. While promising, further development is necessary to address cases in which image quality or positioning issues prevent automated measurement and to reduce reliance on human quality control.

**Level of Evidence:**

Level III.

**Abbreviations**
AI: artificial intelligence
CI: confidence interval
EOS: EOS orthopaedic solution
HKA: hip‐knee‐ankle angle
ICC: intraclass correlation coefficient
JLCA: joint line convergence angle
LAMA: leg axis measurement assistant
LLR: standing long‐leg anteroposterior radiograph
mLDFA: mechanical lateral distal femoral angle
mMPTA: mechanical medial proximal tibial angle
PKA: partial knee arthroplasty
TAA: total ankle arthroplasty
THA: total hip arthroplasty
TKA: total knee arthroplasty

## INTRODUCTION

The hip‐knee‐ankle angle (HKA) is a common parameter that is crucial for the assessment of lower limb alignment in tibia plateau and lower limb fracture as well as for bony deformities and osteoarthritis [6]. Lower limb alignment is essential for surgery planning and postsurgical evaluation in joint arthroplasty, osteotomy and osteosynthesis [12, 14, 15]. Postoperative malalignment is associated with poor function and higher revision rates [4]. Traditionally, these measurements are performed using standing long‐leg anteroposterior radiographs (LLRs) [22]. Due to their significant dependence on the rater's experience [25], these measurements are often time‐consuming, lack reproducibility [10, 18] and must be performed by manually drawing lines and angles on digital radiographs or using specialised software [21].

Currently, artificial intelligence (AI)‐based applications are evolving across every branch of medicine that aim to simplify and accelerate tasks previously performed by human specialists [5]. Recently, the American College of Radiology Data Science Institute identified the measurement of leg length discrepancies in radiographs as a suitable application for AI to enhance medical care [23]. A recent meta‐analysis demonstrated that AI‐based leg‐axis assessment showed excellent agreement with human raters; in addition, the AI‐based assessment was up to ten times faster than the human raters [17].

Various approaches have been used to assess the accuracy of AI‐based leg measurements, ranging from self‐developed solutions [3] to vendor‐provided ones [1]. In the domain of vendor‐provided solutions, the leg axis measurement assistant (LAMA, ImageBiopsy Lab, Vienna, Austria) has been utilised by multiple researchers [13, 19, 21, 23]. However, the Gleamer BoneMetrics (Paris, France) software is also capable of performing various AI‐based leg measurements [9]. Furthermore, its AI algorithm was trained not only on standard radiographs but also on EOS Orthopedic Solution (EOS) images, thereby offering a wide range of possible applications.

A very recent internal study by Gleamer suggested that it has a high rate of accuracy compared to human raters, with a mean absolute error of 0.3° (95% confidence interval [CI] 0.28–0.32) [8]. To our knowledge, no external study has yet confirmed these findings; the recent literature concerning Gleamer has mainly focused on AI‐based fracture recognition [2, 11]. To overcome this limitation, the aim of this study was to assess the accuracy of AI‐based HKA measurements performed by Gleamer BoneMetrics on randomly chosen images. The primary hypothesis was that sufficient agreement between experienced orthopaedic surgeons and the AI‐based software would support its regular usage in clinical practice.

## METHODS

This study was carried out in accordance with the World Medical Association Declaration of Helsinki. Ethical approval was obtained from the ethics committee for this study (231072024‐BO‐E‐RETRO).

### AI software

BoneMetrics, an AI‐based software (version 2.3.0.1, Gleamer, Paris, France) was used for this study. It is a CE‐marked image processing application designed to automate musculoskeletal measurements on standard radiographs and EOS images. The software comprises several convolutional neural networks that allow it to precisely locate landmark points for the computation of various lower limb measurements. Each landmark point is assigned by the algorithm a confidence score from 0 to 100, and only those exceeding a preset threshold of 50 are used for calculations. The software's architecture incorporates a top‐down approach using Detectron2, a streamlined version of HRNet (Lite‐HRNet) and a bottom‐up method. The algorithm was developed using a dataset of over 5000 images from more than 20 European imaging centres that were annotated by 10 specially trained radiographers and radiologists. An expert musculoskeletal radiologist with 14 years of experience meticulously reviewed all of the annotations to guarantee the high quality of the training data, thereby setting a strong foundation for the AI's reliability.

### Data acquisition

The LLRs of 75 randomly selected patients who received a total (*n* = 67; total knee arthroplasty [TKA]) or partial (*n* = 8; partial knee arthroplasty [PKA]) knee arthroplasty in our hospital between October 2023 and June 2024 were retrospectively analysed. None of the pictures from this study was used for the initial training or validation of the AI's algorithm. All of the patients had received an LLR prior to surgery as well as after surgery (Philips DigitalDiagnost, Holland). No images were excluded from this study. The presence of a total hip arthroplasty (THA; *n* = 5) or a total ankle arthroplasty (TAA; *n* = 1) was not a criterion for exclusion.

Two experienced orthopaedic surgeons (MS and NR) with more than ten years of experience each, manually measured the pre‐ and postoperative HKAs twice with a two‐week interval between the measurements (Sectra Workstation IDS7, Version 23.1, Sectra AB, Sweden). There was no interaction between the surgeons. In addition, both surgeons were blinded to the measurement results of the AI algorithm when performing the measurements. Consequently, a total of 150 LLRs were measured by each of the two surgeons and the AI. The AI measurements were also performed twice to assess the intra‐rater reliability of the AI algorithm.

### Statistical analysis

For the statistical analysis, the collected data were provided to an independent researcher (RP) who was uninvolved in the measurement process. The researcher conducted the analysis in a blinded manner, without knowledge of the study's objectives, and was unable to allocate data to a rater or the AI during the calculation process. Descriptive statistics were calculated to summarise the data, including means, standard deviations, medians and interquartile ranges for the continuous variables. Box plots were generated to visually represent the distribution and variability and for illustration of the potential outliers of the measurements conducted by the orthopaedic surgeons and the AI‐based software.

Inter‐ and intra‐rater reliability were evaluated using the intraclass correlation coefficient (ICC) for the continuous variables, with corresponding 95% CIs. The ICC values were interpreted based on established benchmarks: <0.5 = poor, 0.5–0.75 = moderate, 0.75–0.9 = good, and >0.9 = excellent reliability. Additionally, Bland‐Altman plots were used to visually analyse the agreement between the first human rater and the AI pre‐ and post‐surgery. Descriptive statistics were conducted using Microsoft Excel, and the ICC analyses were conducted using IBM SPSS.

## RESULTS

**Figure 1:**
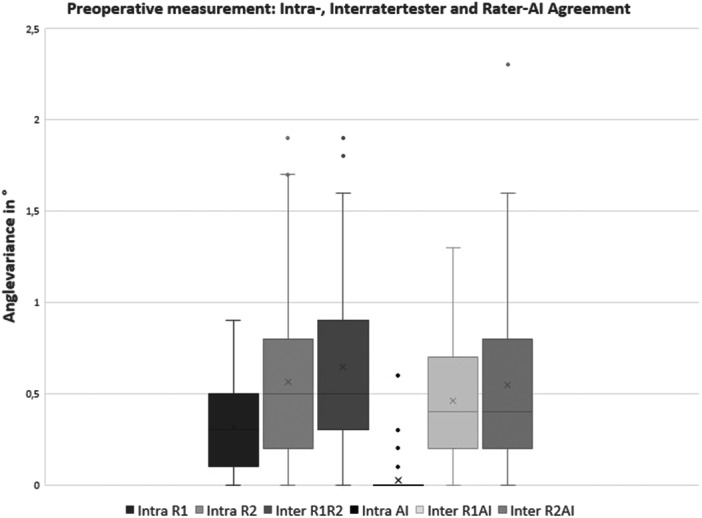
Box plot analysis for preoperative measurements with inter‐ and intra‐rater reliability for human rater 1 (R1), human rater 2 (R2) and the BoneMetrics software. AI, artificial intelligence.

**Figure 2:**
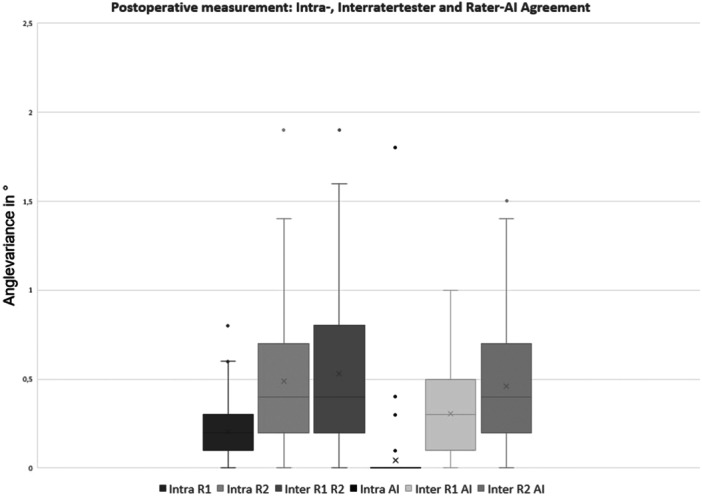
Box plot analysis for postoperative measurements with inter‐ and intra‐rater reliability for human rater 1 (R1), human rater 2 (R2) and the BoneMetrics software. AI, artificial intelligence.

This study included 75 patients who yielded a total of 150 images. The analysis revealed excellent inter‐ and intra‐rater reliability for both orthopaedic surgeons, with ICC values ranging from 0.977 to 0.998. Similarly, the AI‐based software demonstrated a strong correlation with the surgeons' measurements, achieving ICC values of 0.995–0.998 when compared to each human rater. Preoperative AI test‐retest reliability showed perfect agreement (ICC = 1.0), with excellent reliability also observed postoperatively (ICC = 0.996). The ICC, as well as absolute minimal and maximal values are summarised in Table 1. Descriptive analysis indicated minimal measurement error, although perfect agreement was not achieved when the AI was run twice on the same sample. Additionally, a high level of agreement between the AI and the surgeons was observed, which was comparable to the measurement error between the surgeons for both the pre‐ and postoperative assessments (Figures 1 and 2).

**Table 1:** ICC/cronbachs' alpha for intra‐ and interclasscorrelation and absolute minimal and maximal values.

		Pre‐surgery	Post‐surgery	Absolute range in °
Intra‐rater	R1M1_R1M2	0.998	0.996	−0.9 to 0.9
	R2M1_R2M2	0.995	0.977	−1.9 to 1.5
	AIM1_AIM2	1.0	0.966	−0.3 to 0.6
Inter‐rater	R1M1_R2M1	0.995	0.988	−0.8 to 0.8
	R1M1_AIM1	0.998	0.995	−1.9 to 1.4
	R2M1_AIM1	0.996	0.998	−0.3 to 1.8

Abbreviations: AI, artificial intelligence; M, measurment; R, rater.

**Figure 3:**
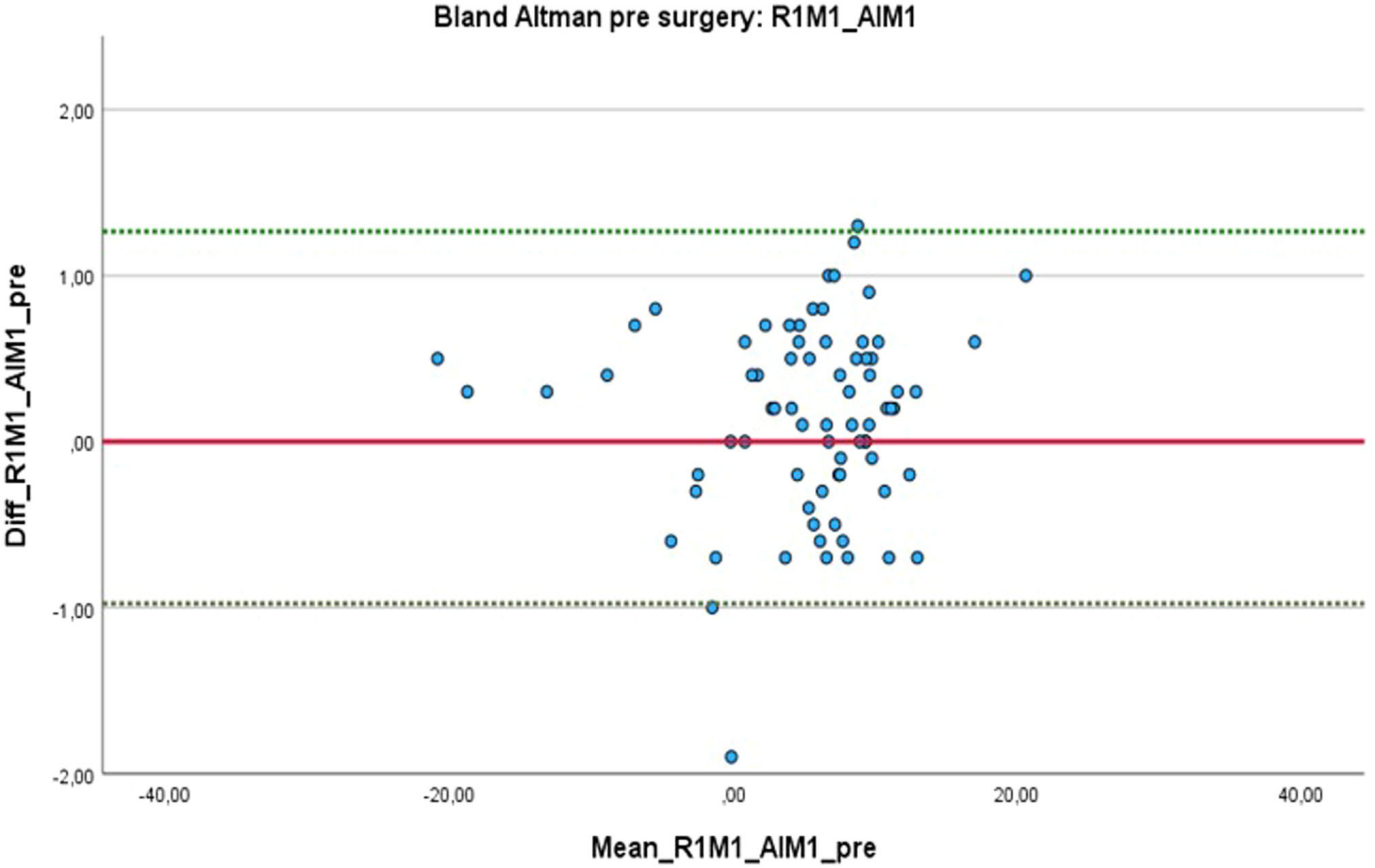
Bland–Altman plot analysis of the preoperative measurements conducted by human rater 1 (R1) and the BoneMetrics software.

**Figure 4:**
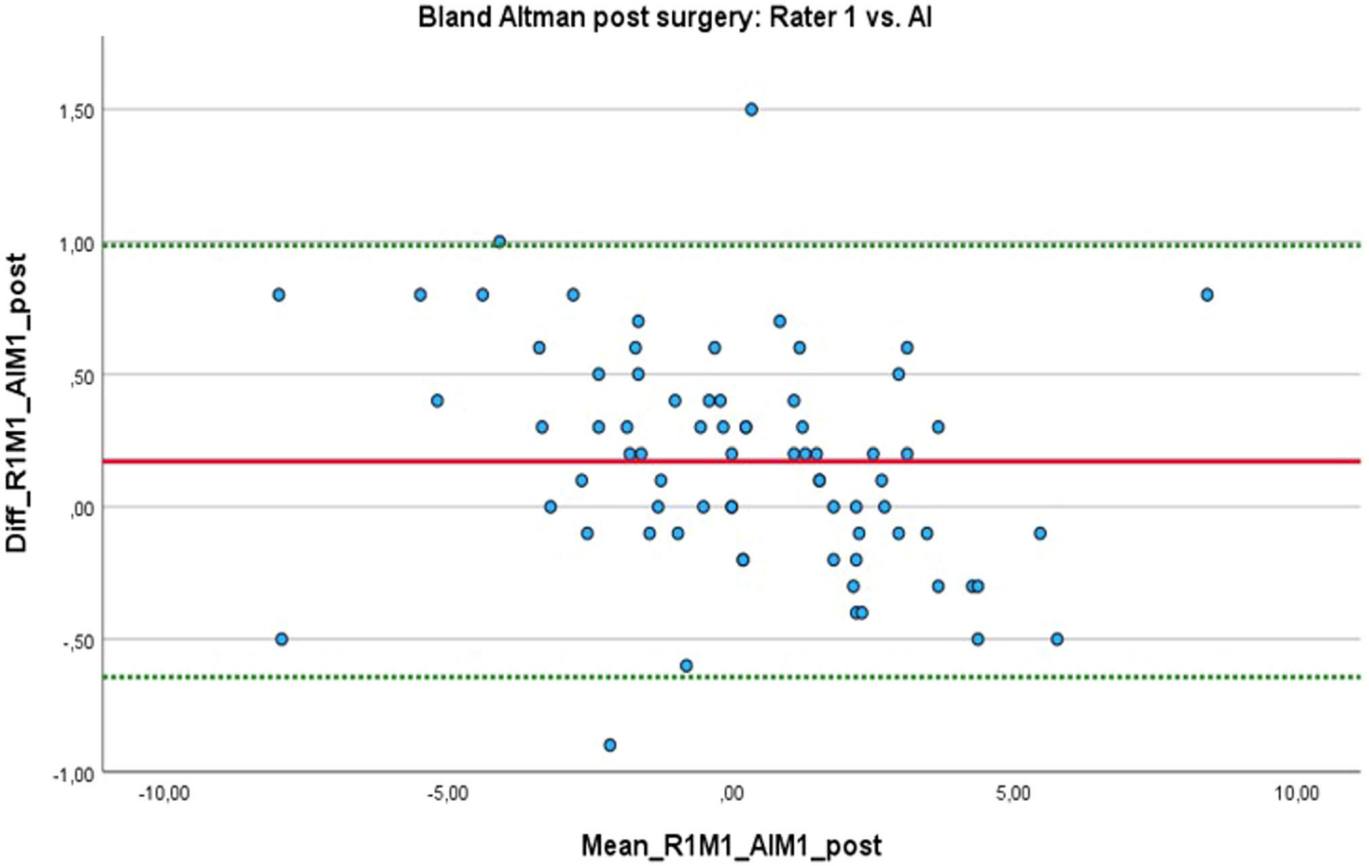
Bland–Altman plot analysis of the postoperative measurements conducted by human rater 1 (R1) and the BoneMetrics software.

The high level of agreement between the first human rater and the AI was visualised using Bland–Altman plots (Figures 3 and 4). Additionally, the pre‐ and postoperative variances between the measurements can be seen in Table 2. It should be noted that the AI was able to perform correct measurements in 98% (*n* = 147) of the cases and failed in 2% (*n* = 3).

**Table 2:** Pre‐ and postoperative measurements including mean values, standard deviations (SD), minimum and maximum value variations with inter‐ and intra‐rater reliability for human rater 1 (R1), human rater 2 (R2) and the BoneMetrics software.

	Intra R1	Intra R2	Inter R1R2	Intra AI	Inter R1AI	Inter R2AI
Preoperative variances between measurments						
Mean	0.31	0.56	0.64	0.02	0.46	0.55
SD	0.26	0.46	0.50	0.09	0.30	0.45
Min	0.00	0.00	0.00	0.00	0.00	0.00
Max	0.90	1.90	1.90	0.60	1.30	2.30
Postoperative variances between measurments						
Mean	0.21	0.49	0.53	0.05	0.31	0.46
SD	0.19	0.40	0.42	0.22	0.25	0.38
Min	0.00	0.00	0.00	0.00	0.00	0.00
Max	0.80	1.90	1.90	1.80	1.00	1.50

Abbreviation: AI, artificial intelligence.

## DISCUSSION

The most significant finding of this study was that AI‐based leg axis assessment of the HKA demonstrated excellent agreement with the experienced human raters. The intra‐ and inter‐rater reliability for both human raters ranged from 0.977 to 0.998. Similar results were observed for the BoneMetrics's intra‐rater reliability (ICC = 1.0 preoperatively and ICC = 0.996 postoperatively) as well as for inter‐rater reliability when the Gleamer BoneMetrics was compared to the human raters (ICC = 0.998).

Comparable findings have been reported by other researchers who used vendor‐provided solutions (including LAMA, ImageBiopsy Lab, Vienna, Austria), which showed excellent ICC values for AI versus human raters [1, 13, 23]. The study by Schwarz et al. showed a mean variation of 0.1° ± 3.4° [19]. Our measurements showed a slightly lower variation with a maximum of 2.3°. The results presented by Simon et al. also similarly demonstrated a variation of up to 2.5° in the Bland–Altman analysis [21]. This suggests that HKA measurements can be reliably performed by the Gleamer BoneMetrics software without any loss of quality because measurement variations of up to two degrees appear to also occur between human raters. Such results offer significant potential for time savings because manual measurements are made unnecessary, thereby allowing radiologists and orthopaedic surgeons to focus on other tasks that are not yet suitable for AI. Additionally, AI‐based solutions may serve as a valuable tool for quality control, particularly for those with limited experience performing these measurements. However, in our study, few measurement outliers were higher than reported by Lassalle et al., who found measurement differences around one degree [8].

We intentionally did not exclude from the study patients with additional implants, such as THA or TAA, so as to assess the potential limitations of the AI algorithm. The presence of THA in five patients did not appear to affect the accuracy of the measurements; both the pre‐ and postoperative measurements were correctly performed by the AI. In three instances, the AI was unable to assess the HKA on the postoperative long‐leg radiographs. In one case, the hip centre was not fully visible on the LLR. In another case, extreme obesity obscured the hip centre on the postoperative LLR. However, the AI was able to perform measurements on the preoperative image, likely due to a slightly different projection of the soft tissue. In a third case, the postoperative image could not be measured by the AI due to the presence of TAA. However, the preoperative image was successfully assessed by the AI. It is hypothesised that the rotational alignment of the radiograph may have negatively influenced the AI's performance in this case.

A few outliers were observed in the study, with standard deviations ranging from 0.09° to 0.5° (mean = 0.02°–0.56°) and a maximum deviation of up to 2.3° in one case. It should be noted that rotational projection can complicate the accurate placement of landmarks, which may explain the few outliers observed, as LLRs performed only a few days postoperatively sometimes are recorded in slight flexion, internal rotation and without full weight bearing due to postoperative pain. During manual measurements, it was also noted that small adjustments to the measuring gauge could lead to significantly different results.

Other studies have assessed the accuracy of AI‐based measurements of various leg axis parameters, such as the mechanical lateral distal femoral angle (mLDFA), the mechanical medial proximal tibial angle (mMPTA), and the joint line convergence angle (JLCA) [7]. However, these measurements are currently not possible with BoneMetrics. Another frequently discussed challenge related to AI‐based measurements is the ‘black‐box’ phenomenon, where only the output is visible, and insight into the measurement and calibration algorithms is limited [20]. To approach this issue, we assessed the intra‐rater reliability of the BoneMetrics, which yielded excellent results. This suggested that the intra‐rater reliability was comparable to or even exceeded that of the experienced human raters in this study.

An unresolved issue that requires further investigation is the problem of human pre‐selection and quality control of images suitable for AI‐based measurements, as malrotation or malpositioning is not accounted for by the AI, which can lead to out‐of‐range measurements [16]. Image quality control, however, is important for correct assessment of the HKA because incorrect projection of LLRs due to postoperative pain and limitation of range of motion often result in incorrect measurements [26]. Although AI allows for fast and accurate measurements across large datasets [24], further advancements in automated quality control are needed to enable these machine‐learning solutions to function independently and reduce reliance on human time and resources [1].

The limitations of the study were as follows. (1) The study was performed at a single centre. (2) The performance of the AI algorithm was measured only in elderly patients prior to TKA or PKA. (3) The assessment of other important parameters, such as the mMPTA, mLDFA or JLCA was not yet possible with BoneMetrics. (4) The study design was retrospective; thus, evaluation of how AI influences everyday clinical practice was not possible.

## CONCLUSION

AI‐based measurement of the HKA with BoneMetrics is an accurate method for preoperative planning as well as for postoperative quality control. The measurement results were similar to those of experienced orthopaedic surgeons in terms of both inter‐rater and intra‐rater reliability. Users must be aware, however, that in some cases automated measurements cannot be performed. The AI‐based recognition of the correct rotation of LLRs is not possible at the present time, but it is crucial to achieve correct automated measurements.

## AUTHOR CONTRIBUTIONS

All authors contributed to the study conception and design. Material preparation and data collection were performed by Mikhail Salzmann and Nikolai Ramadanov. Analysis and statistics were performed by Robert Prill. Extraction of DICOM‐files was performed by Simon Shabo under the supervision of Andreas G. Schreyer. The first draft of the manuscript was written by Mikhail Salzmann and Nikolai Ramadanov. All authors commented on previous versions of the manuscript. All authors read and approved the final manuscript.

## CONFLICT OF INTEREST STATEMENT

The authors declare no conflicts of interest.

## ETHICS STATEMENT

Ethical approval was obtained for this study [231072024‐BO‐E‐RETRO].

## Data Availability

The data is available from the corresponding author on request.
